# Safety and Efficacy of Reduced Dose of Enzalutamide in Patients with Castration-Resistant Prostate Cancer: A Systematic Review

**DOI:** 10.3390/ph18050732

**Published:** 2025-05-16

**Authors:** Zineddine Belabaci, Lucas Mose, Omar El-Taji, Zina Otmani, Zein Alabdin Hannouneh, Issa Mohamad, Thomas Zilli, Osama Mohamad, Nadeem Pervez, Waleed Arafat, Ursula Vogl, Mohamed Shelan

**Affiliations:** 1 Faculty of Medicine, Djillali Liabes University, Sidi Bel Abbes 22000, Algeria; zineddinebelabaci@gmail.com; 2 Department of Radiation Oncology, Inselspital, Bern University Hospital, University of Bern, 3010 Bern, Switzerland; lucas.mose@extern.insel.ch; 3 Genitourinary Cancer Research Group, University of Manchester, The Christie & Salford NHS Foundation Trust, Manchester M20 4BX, UK; omar.el-taji@nhs.net; 4 Faculty of Medicine, Mouloud Mammeri University, Tizi Ouzou 15000, Algeria; otmanizina25@gmail.com; 5 Faculty of Medicine, Al Andalus University for Medical Sciences, Tartus 37444, Syria; zh19@au.edu.sy; 6 Department of Radiation Oncology, King Hussein Cancer Center, Amman 11941, Jordan; imohamad@khcc.jo; 7 Department of Radiation Oncology, Oncology Institute of Southern Switzerland (IOSI), Ente Ospedaliero Cantonale (EOC), 6500 Bellinzona, Switzerland; thomas.zilli@eoc.ch; 8 Faculty of Biomedical Sciences, Università della Svizzera Italiana, 6900 Lugano, Switzerland; 9 Faculty of Medicine, University of Geneva, 1211 Geneva, Switzerland; 10 Department of Genito-Urinary Radiation Oncology, MD Anderson Cancer Center, Houston, TX 77030, USA; omohamad@gmail.com; 11 Department of Internal Medicine, College of Medicine and Health Sciences (CMHS), UAE University, Abu Dhabi P.O. Box 15551, United Arab Emirates; nadeemp@yahoo.com; 12 Department of Clinical Oncology, Faculty of Medicine, Alexandria University, Alexandria 21526, Egypt; waleed_o_arafat@yahoo.com; 13 Department of Medical Oncology, Oncology Institute of Southern Switzerland (IOSI), EOC, 6500 Bellinzona, Switzerland; ursula.vogl@eoc.ch

**Keywords:** castration-resistant prostate cancer, enzalutamide, dose, efficacy, adverse events

## Abstract

**Objective:** To review the efficacy and safety of reduced dose compared to standard dose Enzalutamide treatment for patients with castration-resistant prostate cancer (CRPC). **Methods:** PubMed, Scopus, Web of Science, and Cochrane databases were searched for randomized controlled trials and cohort studies reporting the use of Enzalutamide in reduced and standard doses in patients with castration-resistant prostate cancer. Searches were limited to articles published in the English language. Outcome assessments included progression-free survival (PFS), overall survival (OS), adverse events, and serum prostate-specific antigen (PSA) response. **Results:** Ten studies met the inclusion criteria, including 2481 patients treated with Enzalutamide. Seven studies were retrospective cohorts, two were prospective trials, and one was a prospective cohort. No consistent relationship was identified between OS and PFS and the Enzalutamide dosage. Reduced doses of Enzalutamide decreased the incidence of adverse events, particularly among elderly patients. **Conclusions:** This systematic review suggests that reduced doses of Enzalutamide in CRPC may maintain therapeutic efficacy in selected patients while improving tolerability. However, inconsistent findings and methodological limitations highlight the need for prospective randomized trials to define optimal and individualized dosing strategies.

## 1. Introduction

Prostate cancer is the second most commonly diagnosed cancer in men and the fifth leading cause of cancer-related mortality worldwide, with over 1.5 million new cases and 397,000 deaths reported in 2022 [1]. Despite advancements in primary treatment, approximately 20–50% of patients initially treated with radical prostatectomy or radiotherapy with curative intent will experience biochemical recurrence, necessitating subsequent androgen deprivation therapy (ADT) [2,3]. Additionally, around 10% of patients present with metastatic disease at the time of diagnosis and typically receive ADT as a first-line treatment [4,5]. Castration-resistant prostate cancer (CRPC), a state in which the disease progresses despite low testosterone levels, often develops within three years of initiating ADT [6]. Since the approval of docetaxel for CRPC in 2004 [7], the therapeutic landscape has significantly evolved. New treatment options have emerged, including androgen receptor pathway inhibitors (ARPIs) such as apalutamide, Enzalutamide, darolutamide, and Abiraterone acetate, as well as cabazitaxel and radionuclide therapies, including 177Lu-PSMA and radium-223 [8,9]. These advances have expanded the range of available treatments, improving clinical outcomes for patients with advanced prostate cancer.

Enzalutamide is a highly potent androgen receptor pathway inhibitor (ARPI) that exerts its effects on tumor cells through multiple mechanisms. It disrupts androgen receptor (AR) nuclear translocation, impairs AR binding to DNA, and inhibits the recruitment of coactivators essential for transcriptional activity, ultimately inducing apoptosis. Compared to other ARPIs, Enzalutamide exhibits a higher binding affinity to the AR, a critical driver of castration resistance and tumor progression in prostate cancer [6,10]. This efficacy has been supported by multiple prospective clinical trials demonstrating the benefits of Enzalutamide in patients with CRPC. Notably, the PREVAIL and AFFIRM trials showed a significant improvement in overall survival (OS), with PREVAIL focusing on chemotherapy-naïve patients and AFFIRM targeting those previously treated with chemotherapy [11,12]. Similarly, PROSPER reported longer OS in non-metastatic CRPC compared to placebo [13]. Additionally, in both STRIVE (chemotherapy-naïve) and TERRAIN (non-metastatic prostate cancer) trials, Enzalutamide showed significant improvements in PFS and OS compared to bicalutamide, with notable enhancements in both PFS and OS outcomes [14,15]. Recent EMBARK findings highlight Enzalutamide’s potential in high-risk localized prostate cancer, showing significant improvements in metastasis-free survival (MFS) when combined with ADT or used as monotherapy, compared to ADT alone, with a safety profile consistent with prior studies [16].

In the aforementioned trials, the recommended standard dose for Enzalutamide in CRPC is 160 mg per day [12,13,14]. However, up to 90% of patients experience adverse events (AEs), such as fatigue, back pain, and hot flashes, which can lead to treatment interruptions or dose reductions [12,13,14,15,17]. This might lead to decreased treatment efficacy, with a considerable proportion of patients having to reduce or interrupt treatment due to side effects [18,19]. There are, however, data suggesting that AR saturation could be achieved with lower doses of Enzalutamide, with little to no loss of efficacy. This raises the question of whether dose reduction in CRPC patients could reduce side effects while maintaining antitumoral efficacy [10,20].

Given the sparse literature, the aim of this work was to systematically review and summarize the available literature on the efficacy and safety of the reduction in Enzalutamide in patients with CRPC disease.

## 2. Materials and Methods

### 2.1. Methods

This systematic review was conducted adhering to the Cochrane Handbook of Systematic Reviews of Interventions [21] at each step, and following the Preferred Reporting Items for Systematic reviews and Meta-Analysis (PRISMA) statement [22]. The review protocol was registered at PROSPERO (CRD42024567029).

### 2.2. Study Selection

A comprehensive search of four databases (PubMed, Scopus, Web of Science, and Cochrane databases) was conducted from inception to July 2024 utilizing a well-constructed search strategy (Supplementary Materials, Table S3).

Publications were included for review if they were randomized controlled trials or cohort studies that reported on reduced and standard doses of Enzalutamide in adult patients diagnosed with CRPC. Searches were limited to studies published in English. Publications were excluded if they were reviews, editorials, commentaries, case reports, meeting abstracts, or if they presented findings from animal studies. Outcome assessments included prostate-specific antigen (PSA) response, progression-free survival (PFS), OS, and AEs.

The articles identified through the literature search were imported into EndNote software version 21 (Clarivate Analytics), and duplicates were removed. The screening was conducted independently by two authors in a two-step process. Initially, the retrieved references were assessed for eligibility based on title and abstract. Subsequently, the studies deemed eligible were further evaluated through full-text review. Any disagreements regarding inclusion or exclusion were resolved through discussion or by the senior author, as necessary.

### 2.3. Data Extraction

Study characteristics of the included studies, encompassing article information (e.g., first author, publication year, and country), study design, Enzalutamide dose, population, number of patients, outcome measures, and key findings were extracted by two authors and summarized.

### 2.4. Quality Assessment

Two authors conducted the quality and risk of bias assessment independently. Any disagreements were discussed and resolved by mutual agreement or by the senior author. The Newcastle–Ottawa Scale (NOS) was utilized to assess the quality of the observational studies [23]. The Cochrane tool for assessing risk of bias in randomized trials (RoB2) was utilized to assess the risk in randomized trials [24].

### 2.5. Analysis of Results

Due to the heterogeneity in endpoints, patient characteristics, and treatment regimens, a meta-analysis for toxicity, PSA-PFS, or OS was not performed. Instead, the studies were analyzed using a narrative synthesis approach.

## 3. Results

### 3.1. Evidence Synthesis

**Figure 1:**
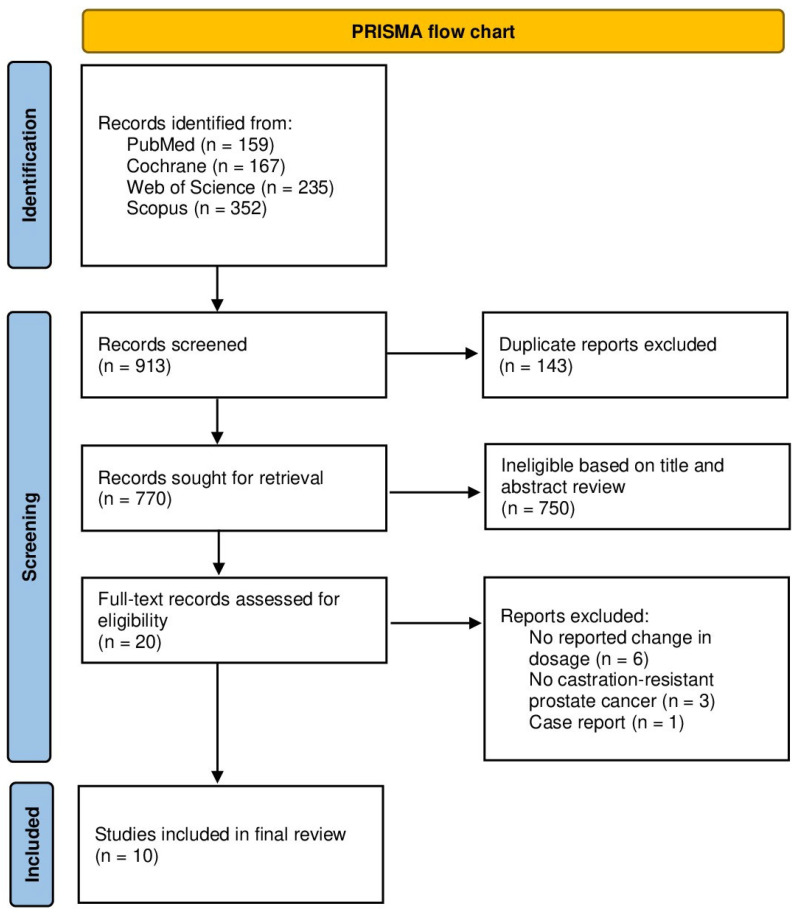
PRISMA flow chart.

Following the retrieval of 913 records and the removal of 143 duplicates, a total of 770 records underwent title and abstract review. This resulted in the exclusion of 750 records. The remaining 20 publications were assessed for inclusion eligibility via full-text review. Consequently, a total of 10 studies were included in the final review. A PRISMA flow chart summarizing the process is displayed in Figure 1.

### 3.2. Characteristics of the Included Studies

A total of ten studies were included in this systematic review, with a sample size of 2481 patients. Five studies were conducted in Japan, two each in the USA and France, and one in the Netherlands. The median age of patients in the reported studies ranged from 68 to 85 years. Nine studies reported on patients diagnosed with CRPC only, while one study included both patients in the CRPC and hormone-sensitive setting. Minimal reported dose varied between studies, with a minimal daily dose of 30 mg per day, with the reference dose being 160 mg Enzalutamide per day. The baseline characteristics are summarized in Table 1.

### 3.3. Progression-Free Survival (PFS) and Overall Survival (OS)

The relationship between Enzalutamide dosing and progression-free survival (PFS) varied across studies. Yamada et al. (2022) reported significantly shorter PFS in patients receiving reduced doses (<160 mg/day) compared to the standard 160 mg/day dose (7.2 months vs. 12.1 months; *p* = 0.037), although overall survival (OS) remained unaffected [25]. Similarly, Tsuzuki et al. (2021) observed higher PSA response rates (*p* = 0.02) and greater PSA declines (*p* = 0.03) in patients treated with the standard dose. However, only a trend toward longer PFS was noted (8 months vs. 6 months; *p* = 0.1) [18]. Freedland et al. (2021) found a significant correlation between lower relative dose intensity (<80%) and shorter PSA PFS (*p* < 0.01), emphasizing the importance of maintaining dose adherence for optimal outcomes [26] (Table 2).

Contrasting these findings, Vinh-Hung et al. (2020, 2022) [27,28] found no significant differences in PFS or OS between patients receiving doses ≤80 mg/day and those on the standard 160 mg/day dose. However, reduced doses were associated with higher PSA responses and improved longevity in specific subgroups, such as elderly or frail patients [27,28]. Similarly, Boerrigter et al. (2024) reported no significant PFS or OS differences between patients receiving 120 mg/day and those on 160 mg/day, with efficacy maintained in the reduced-dose group [20]. Hori et al. (2020) also observed no relationship between PSA response and Enzalutamide doses ranging from 80 to 160 mg/day [29].

Exploring alternative dosing strategies, Miura et al. (2021) demonstrated that a dose-escalation regimen, starting at 80 mg/day and gradually increasing to 160 mg/day, resulted in a longer median time to treatment failure (18.0 months vs. 10.4 months) with no significant PFS disadvantage compared to the standard dose from treatment initiation [8].

**Table 1:** Characteristics of included studies.

Study	Country	Study Design	Dose	Age (in Years)	Population	No. of Patients
Scher et al., 2010 [10]	USA	Phase I–II study trial	Enzalutamide 30–600 mg	68 (44–93)	mCRPC	140
Terada et al., 2016 [30]	Japan	Retrospective multi-institutional cohort study	Enzalutamide standard doses of 160 mg/day and lower doses (80–120 mg/day)	69 (46–90)	CRPC	345 Standard dose: 266 Reduced dose: 79
Hori et al., 2020 [29]	Japan	Real-world cohort of Japanese patients with CRPC	Enzalutamide 160 mg/day vs. 80, 120 mg/day	79 (69–91)	CRPC	13 Standard dose: 4 Reduced dose: 9
Vinh-Hung et al., 2020 [27]	France (Martinique)	Retrospective cohort	Enzalutamide 160 mg/day vs. ≤80 mg/day	Standard dose: 80.1 (74.7–90.3) Reduced dose: 84.3 (74.9–93.8)	mCRPC	59 Standard dose: 43 Reduced dose: 16
Miura et al., 2021 [8]	Japan	Retrospective cohort	Enzalutamide standard dose (started at 160 mg) or dose-escalation (started at 80 mg, followed by dose escalation)	Standard dose: 70.5 (63−84) Reduced dose: 78 (64−93)	CRPC	107 Standard dose: 17 Dose-escalation: 90
Freedland et al., 2021 [26]	USA	Retrospective longitudinal cohort study of US Veterans Health Administration data	Enzalutamide reduced dose <80%	74.5	mCRPC	1375
Tsuzuki et al., 2021 [18]	Japan	Retrospective cohort	Enzalutamide 160 mg/day vs. 40–120 mg/day	Standard dose: 76 (69–80) Reduced dose: 81 (76–84) (median, IQR)	CRPC	233 Standard dose: 190 Reduced dose: 43
Vinh-Hung et al., 2022 [28]	France (Martinique)	Retrospective cohort	Enzalutamide 160 mg/day vs. ≤80 mg/day	Standard dose: 76.5 (52.6–90.3) Reduced dose: 81.9 (58.4–93.8)	mCRPC	111 Standard dose: 79 Reduced dose: 32
Yamada et al., 2022 [25]	Japan	Retrospective cohort	Enzalutamide 160 mg/day or dose reduction from 30 to 150 mg	75 (70–82) (median, IQR)	CRPC	105 Standard dose: 77 Reduced dose: 28
Boerrigter et al., 2024 [20]	The Netherlands	Multicentric open-label, randomized clinical trial	Enzalutamide 160 mg/day vs. 120 mg/day	Standard dose: 80 (68–88) Reduced dose: 80 (69–90)	mCRPC (48) mHSPC (4)	52 Standard dose: 27 Reduced dose: 25

Abbreviations: CRPC: castration-resistant prostate cancer; mCRPC: metastatic castration-resistant prostate cancer; mHSPC: metastatic hormone-sensitive prostate cancer; and IQR: interquartile range. Data are presented as median (range) for age.

**Table 2:** Enzalutamide: efficacy and toxicity findings.

Study	Summary of Methods	Efficacy Findings	Toxicity Findings
Scher et al., 2010 [10]	Phase I–II trial evaluating doses between 30 and 600 mg/day to determine maximum tolerated dose and antitumor effects.	PSA reduction ≥50% in 56% of patients. Median time to radiological progression: 47 weeks.	Grade 3–4 fatigue: 11% (dose-dependent). Resolved after dose reduction.
Terada et al., 2016 [30]	Retrospective cohort study comparing patients on standard dose (160 mg/day) vs. reduced dose (<160 mg/day).	PSA reduction ≥50% in 57% of patients. Median PSA progression-free survival: 163 days.	AEs (fatigue, appetite loss): 49%. Discontinuation due to AEs: 18%. Older age and lower doses are associated with fewer AEs.
Hori et al., 2020 [29]	Real-world study comparing efficacy and safety in CRPC patients on doses ranging from 80 to 160 mg/day.	PSA decrement ≥50% in 92% of patients.	AEs were mild (>20%). No significant safety concerns in Japanese CRPC patients.
Vinh-Hung et al., 2020 [27]	Retrospective study comparing patients on standard dose (160 mg/day) and low dose (<80 mg/day).	PSA reduction ≥50% in 67% (low dose) vs. 45% (standard dose). Median PFS: 11.2 months (low dose) vs. 11.9 months (standard dose).	Low-dose is associated with reduced toxicity in elderly, poor-performance patients.
Miura et al., 2021 [8]	Patients were divided into standard-dose (160 mg/day) and dose-escalation (80 mg/day, gradually increasing to 160 mg/day) groups.	Median TTF: 10.4 months (standard dose) vs. 18.0 months (dose escalation).	Grade ≥3 AEs: 23.5% (standard dose) vs. 6.7% (dose escalation). AEs (any grade): 88.2% (standard dose) vs. 63.3%. Discontinuation due to AEs: 35.3% (standard dose) vs. 12.2%.
Tsuzuki et al., 2021 [18]	Retrospective analysis comparing standard dose (160 mg/day) and reduced dose (40–120 mg/day) in CRPC patients.	PSA response rate: −66.3% (reduced dose) vs. −87.4% (standard dose).	AE incidence: 22.6% (reduced dose) vs. 34.4% (standard dose). No significant difference.
Freedland et al., 2021 [26]	Large retrospective analysis using US Veterans Health Administration data to assess the impact of relative dose intensity on outcomes.	Dose reductions (RDI <80%) are associated with an 8.8% higher risk of PSA progression.	Dose reductions increased PSA progression risk. Patient adherence is recommended.
Vinh-Hung et al., 2022 [28]	Subgroup analysis of patients receiving standard (160 mg/day) vs. low-dose (<80 mg/day) therapy.	OS and PFS did not differ between low and standard doses. The low dose showed better longevity (5.3 years).	Low dose is associated with fewer AEs, particularly in patients with comorbidities and poor performance.
Yamada et al., 2022 [25]	Retrospective study comparing outcomes in patients on standard (160 mg/day) vs. reduced dose (<160 mg/day).	Median PFS: 12.1 months (standard dose) vs. 7.2 months (reduced dose). Reduced doses showed inferior oncological outcomes.	Dose reduction increased the risk of disease progression but did not affect OS.
Boerrigter et al., 2024 [20]	Prospective trial comparing reduced dose (120 mg/day) and standard dose (160 mg/day) in prostate cancer patients.	Reduced dose maintained efficacy. No significant interference with efficacy endpoints.	The reduced dose is associated with less fatigue, cognitive, and depressive symptoms. The standard dose showed worsening side effects over 24 weeks.

TTF: Time to treatment failure; AE(s): adverse event(s); PSA: prostate-specific antigen; PFS: progression-free survival; OS: overall survival; CRPC: castration-resistant prostate cancer; and RDI: relative dose intensity.

### 3.4. Toxicity and Treatment Adherence

The toxicity of Enzalutamide was dose-dependent, as reported by multiple studies. Scher et al. (2010) highlighted fatigue as the most common adverse event (AE) across all doses, with higher-grade fatigue observed at higher doses [10]. Similarly, Terada et al. (2016) identified the standard dose (160 mg/day) as a significant predictor of AE development (*p* < 0.01), with older patients experiencing fewer AEs at reduced doses [30]. Miura et al. (2021) reported significantly fewer grade ≥3 AEs (6.7% vs. 23.5%) and overall AEs (63.3% vs. 88.2%) in patients on a dose-escalation regimen compared to those starting with the standard dose, along with lower discontinuation rates (12.2% vs. 35.3%) [8] (Table 2).

Boerrigter et al. (2024) noted reduced fatigue and stable cognitive and depressive symptoms in patients on 120 mg/day compared to those on 160 mg/day, where worsening symptoms were reported [20]. Tsuzuki et al. (2021) [18] found no significant differences in AE incidence between patients receiving 80–120 mg/day and those on the standard dose (22.6% vs. 34.4%), while Miura et al. observed a trend toward fewer treatment discontinuations in the dose-escalation group (*p* = 0.028) [8].

Vinh-Hung et al. (2020, 2022) [27,28] and Hori et al. (2020) confirmed that lower doses were associated with reduced toxicity, particularly in elderly or frail patients, without compromising safety [29]. Conversely, Freedland et al. (2021) emphasized that dose reductions below 80% relative dose intensity increased the risk of PSA progression (HR = 1.258; *p* = 0.003), highlighting the trade-off between reduced toxicity and disease control [26] (Table 2).

### 3.5. Quality Assessment

According to NOS, four of the included studies were considered high quality and four were considered moderate quality because some outcomes were not reported. Utilizing the RoB2 tool, one randomized trial was categorized with “some concerns” due to the quality of outcome measurement. The results of the quality and risk of bias assessment are summarized in (Supplementary Materials, Tables S1 and S2).

## 4. Discussion

To the best of our knowledge, this is the first systematic review evaluating the safety and efficacy of Enzalutamide dose reduction in patients diagnosed with CRPC. In addition to being a standard of care in mHSPC and its emerging role in non-metastatic HSPC, Enzalutamide represents an effective treatment for patients with both non-metastatic and metastatic CRPC, including those who are chemotherapy-naive and those post-chemotherapy [11,12,13,31]. Many patients, however, experience side effects that may necessitate dose reduction or treatment discontinuation [11,12,18,19]. Most of the studies included in this systematic review indicate that reducing the dose of Enzalutamide could lead to a decrease in side effect rates with minimal to no loss of efficacy. However, the majority of these studies are retrospective, and there is variability in the cohorts and dose reduction protocols across studies. Consequently, these results should be interpreted with caution.

In several studies, PSA response was used as a marker of therapeutic efficacy in patients with CRPC treated with an altered Enzalutamide dose. In a cohort study of 59 patients, Vinh-Hung et al. observed no significant difference in PSA response rates at 12 weeks between patients receiving daily doses of <80 mg or 160 mg of Enzalutamide [27]. However, in a follow-up analysis of 111 patients, the same group reported higher PSA response rates and declines in those treated with less than 80 mg daily [28]. These findings suggest that lower doses may achieve similar efficacy in certain patient subgroups. Yet the impacts of prior treatments, which are known to reduce PSA response in CRPC, present a potential confounding factor, as patients in the lower-dose cohort had undergone fewer prior systemic therapies [10,28]. In contrast, Tzusuki et al. reported superior PSA response rates and declines with 160 mg of Enzalutamide daily compared to lower doses (40–120 mg) [18]. Notably, no significant differences in prior docetaxel use were identified between the dose groups. However, the observed differences may be influenced by patient performance status, as those receiving the higher dose had better baseline performance, a factor known to impact PFS [18,30]. These findings are consistent with the prospective trial by Scher et al., which demonstrated a dose-dependent PSA response up to 150 mg daily, beyond which no additional benefit was observed [10]. These studies highlight the complex relationship between dose, patient characteristics, and PSA response, underscoring the need for further investigation into optimizing Enzalutamide dosing to balance efficacy and tolerability.

While evidence exists correlating PSA response with clinical outcomes in mHSPC, no studies have definitively demonstrated an association between PSA response and PFS in CRPC. The literature presents conflicting data regarding PFS outcomes post-dose reduction, with some investigations reporting decreased intervals while others demonstrate equivalent durations. In a cohort study of 162 chemotherapy-naive patients, Yamada et al. observed significantly prolonged PFS in those receiving standard-dose versus reduced-dose therapy. However, this finding warrants careful interpretation, as patients requiring dose reductions exhibited significantly elevated baseline PSA levels, an established negative prognostic indicator for PFS [25,26,30]. In contrast, Vinh-Hung et al. reported no significant inter-group differences in baseline PSA levels between standard- and reduced-dose cohorts [27]. However, their analysis of 16 patients receiving reduced-dose Enzalutamide, with a median follow-up duration of 9.9 months, potentially lacks sufficient statistical power to draw definitive conclusions [27].

In the largest retrospective analysis to date, Freedland et al. examined 1375 patients and demonstrated a significantly elevated hazard ratio for PSA progression in patients receiving less than 80% of the prescribed dose [26]. However, the retrospective design precludes identification of dose reduction rationales, potentially confounding the observed shorter PFS with factors such as poor performance status or medication adherence [26,30]. The sole prospective investigation by Boerritger et al. randomized 52 frail patients with predominantly metastatic castration-resistant prostate cancer to receive either 160 mg or 120 mg of Enzalutamide daily [20]. At median follow-up of 17.7 months, median PFS was 8.8 months and 22.8 months for reduced and standard doses, respectively, though statistical significance was not achieved (*p* = 0.14) [20]. This PFS differential could suggest inferior outcomes with dose reduction or reflect the higher baseline PSA levels in the reduced-dose cohort [20,25]. Notably, overall survival remained comparable between cohorts (34 versus 31.4 months for reduced and standard doses, respectively; *p* = 0.97) [20]. This finding aligns with multiple studies demonstrating no significant overall survival differences between reduced and standard Enzalutamide dosing [25,27,28]. However, Boerrigter et al. acknowledged insufficient statistical power to establish non-inferiority between dosing regimens [20].

Regarding toxicity, multiple studies demonstrate significantly decreased adverse event frequency with dose reduction, correlating with improved treatment adherence [8,10,20,30]. This finding is supported by the observation of Iguchi et al., where a reduced dose of Enzalutamide led to a lower serum level of Enzalutamide and its active metabolite *N*-desmethyl enzalutamide with concurrent adverse event resolution [32]. Among the studies included in this systematic review, only one study failed to achieve statistical significance in adverse event reduction; however, a favorable trend with dose reduction was reported [18].

Among various AEs, such as hypertension, appetite loss, nausea, cognitive impairment, and depression, fatigue is the most frequently reported, affecting up to 34% of patients [10,12,18,19,20,30]. Scher et al. identified fatigue as a dose-dependent AE, typically emerging around four weeks after treatment initiation, when Enzalutamide reaches steady-state plasma levels, and resolving within weeks after dose reduction [10]. Similarly, in the prospective trial by Boerrigter et al., fatigue occurred significantly less often in patients undergoing dose reductions, supporting a correlation between reduced dosing and improved tolerability [20].

Similar to fatigue, other AEs also appear to be dose-dependent. Both retrospective and prospective studies have reported significantly lower rates of any AEs in patients receiving lower doses of Enzalutamide compared to those on standard doses [10,18,20,30]. Interestingly, lower AE rates and higher treatment adherence were reported when Enzalutamide treatment was initiated with 80 mg, escalating to 160 mg daily within a month. However, this approach was only compared to a small cohort of patients who started with 160 mg, and further evaluation in larger prospective cohorts is needed to confirm these results [8].

The therapeutic strategy of ARPI dose reduction to optimize the efficacy–toxicity profile has previously been investigated. Szmulewitz et al. conducted an international, randomized phase II clinical trial investigating the combination of low-dose Abiraterone with low-fat meal consumption to enhance drug bioavailability [33]. The study demonstrated non-inferiority of low-dose Abiraterone compared to the standard fasting dose in CRPC patients, as measured by PSA response and PFS, while maintaining comparable adverse event profiles [33]. These findings were also retrospectively replicated in the HSPC setting [34].

Despite being the first systematic review reporting on Enzalutamide dose reduction in CRPC, several methodological limitations warrant consideration. The predominantly retrospective nature of included studies introduces potential selection bias, while heterogeneity in dose reduction protocols and PFS definitions complicates inter-study comparisons. Additionally, limited sample sizes in several investigations may preclude adequate statistical power, particularly for OS analyses.

The ongoing randomized phase II ANZadapt trial (NCT05393791) employs an adaptive treatment strategy, investigating intermittent administration of Abiraterone or Enzalutamide in metastatic CRPC patients. This protocol, modeled after the EMBARK trial methodology, initiates treatment until achieving a 50% PSA reduction, followed by treatment suspension and subsequent re-initiation upon PSA return to baseline. With time to treatment failure as the primary endpoint and OS and AEs as secondary endpoints, this trial may provide clearer evidence regarding optimal dosing strategies.

## 5. Conclusions

This systematic review represents the first comprehensive evaluation of Enzalutamide dose reduction in patients with CRPC. The current evidence suggests that lower Enzalutamide doses may preserve therapeutic efficacy, particularly in selected patient subgroups, while significantly reducing adverse event rates and improving tolerability. However, findings across studies remain inconsistent due to heterogeneous methodologies, retrospective designs, and limited statistical power. Future research, including a prospective randomized trial, is necessary to validate these findings and establish optimal dosing strategies and personalized approaches for patients.

## Data Availability

All the data are available in the manuscript; any additional information can be provided by contacting the corresponding author.

## Supplementary Materials

The following supporting information can be downloaded at: https://www.mdpi.com/article/10.3390/ph18050732/s1, Table S1: Newcastle Ottawa Quality Assessment Scale (NOS) for cohort studies. Table S2: RoB2 and ROBINS-I assessment forms for clinical trials. Table S3: Search Strategy for Meta-Analysis. The PRISMA checklist.

## Abbreviations

ADT	Androgen deprivation therapy
AEs	Adverse events
AR	Androgen receptor
ARPI	Androgen receptor pathway inhibitor
CI	Confidence Interval
CRPC	Castration-resistant prostate cancer
CV	Cardiovascular
HSPC	Hormone-sensitive prostate cancer
HR	Hazard ratio
IQR	Interquartile range
MFS	Metastasis-free survival
NOS	Newcastle–Ottawa Scale
OS	Overall survival
PFS	Progression-free survival
PRISMA	Preferred Reporting Items for Systematic reviews and Meta-Analyses
PSA	Prostate-specific antigen
RoB	Risk of bias
RoB2	Risk of bias 2 tool
RCT	Randomized controlled trial
ENZA	Enzalutamide
